# High expression of *RIPK2* is associated with Taxol resistance in serous ovarian cancer

**DOI:** 10.1186/s13048-022-00986-2

**Published:** 2022-04-27

**Authors:** Yuqing Shen, Hui Lin, Kelie Chen, Wanzhong Ge, Dajing Xia, Yihua Wu, Weiguo Lu

**Affiliations:** 1 Women’s Reproductive Health Laboratory of Zhejiang Province, Women’s Hospital School of Medicine Zhejiang University, Hangzhou, 310006 China; 2 Department of Gynecologic Oncology of Women’s Hospital School of Medicine Zhejiang University, Hangzhou, 310058 Zhejiang China; 3 Department of Toxicology of School of Public Health, and Department of Gynecologic Oncology of Women’s Hospital School of Medicine Zhejiang University, Hangzhou, 310058 Zhejiang China; 4 Division of Human Reproduction and Developmental Genetics, Women’s Hospital School of Medicine Zhejiang University, Hangzhou, 310058 Zhejiang China; 5 Institute of Genetics and Department of Genetics School of Medicine Zhejiang University, Hangzhou, 310058 Zhejiang China; 6 Cancer Center, Zhejiang University, Hangzhou, 310058 Zhejiang China

**Keywords:** Serous ovarian cancer, Taxol resistance, *RIPK2*, Bioinformatics, Immune infiltration

## Abstract

**Background:**

Taxol resistance in serous ovarian cancer is responsible for its poor prognosis, yet the underlying mechanism is still poorly understood. Thus, we probed the mechanism of Taxol resistance in serous ovarian cancer with multiple bioinformatic methods to provide novel insights into potential therapies.

**Methods:**

The differentially expressed genes (DEGs) in Taxol-sensitive and Taxol-resistant cell lines and their relationship with the overall survival (OS) and progression-free interval (PFI) of ovarian cancer patients were analyzed using gene expression datasets from the Cancer Genome Atlas (TCGA) and Gene Expression Omnibus (GEO). The role of receptor interacting serine/threonine kinase 2 (*RIPK2*) was validated via identification of its coexpressed genes, functional analysis and generation of a protein-protein interaction (PPI) network. The single sample gene set enrichment analysis (ssGSEA) was used to explore immune infiltration, and genomic alterations of *RIPK2* were also analyzed via cBio Cancer Genomics Portal (cBioProtal).

**Results:**

*RIPK2* was highly expressed in Taxol resistant ovarian cancer cell lines, and its high expression was also linked with shorter OS and PFI in serous ovarian cancer patients. The PPI network analysis and pathway analysis demonstrated that *RIPK2* might participate in the positive regulation of NF-κB transcription factor activity. *RIPK2* expression was related to tumor microenvironment alterations, which might participate in the formation of Taxol resistance.

**Conclusions:**

Our studies suggested that high expression of *RIPK2* is related to Taxol resistance in serous ovarian cancer, and that *RIPK2* induces Taxol resistance through NOD1/RIPK2/NF-κB inflammatory pathway activation and tumor microenvironment changes.

**Supplementary Information:**

The online version contains supplementary material available at 10.1186/s13048-022-00986-2.

## Introduction

Serous ovarian cancer (SOC) accounts for 70% of all ovarian cancers and is known as the most common subtype of ovarian cancer [1]. SOC includes high-grade serous ovarian cancer (HGSOC) and low-grade serous ovarian cancer (LGSOC), and HGSOC has the highest mortality [2]. Ovarian cancer is conventionally treated with surgery and paclitaxel/carboplatin combination chemotherapy [3]. Although patients may initially respond well to chemotherapy, the 5-year survival rate is still low because of late-stage diagnosis, disease heterogeneity and drug resistance [4].

Taxol is recommended along with platinum as the first-line chemotherapeutic agent against ovarian cancer [5]. However, the majority of patients suffer from disease recurrence and chemoresistance during treatment. Recent studies have revealed that Taxol resistance may be caused by a series of modifications, including tumor microenvironment changes, pharmacokinetic alterations, signaling pathways changes, P-glycoprotein (P-gp)upregulation, tubulin dynamic alterations, *β*-tubulin gene or *β*-tubulin isotype mutations and apoptotic change [6]. Alterations in gene expression levels also play a significant role in the development of Taxol resistance. For example, high expression of tubulin beta 3 class III (*TUBB3*) and low expression of salt inducible kinase (*SIK2*), polo-like kinase 2 (*PLK2*) or spleen tyrosine kinase (*SYK*) restore the paclitaxel sensitivity of ovarian cancer cells [7–9]. Nevertheless, the mechanisms of Taxol resistance in ovarian cancer are more poorly understood, and more attention to these topics should be given.

Bioinformatics analysis is a rapidly advancing method used widely in cancer-related studies, the application of which has caused the emergence of a great number of studies focusing on cancer chemoresistance and recurrence-related genes. Radosław Januchowski et al. [10] used microarray analysis and observed upregulation of ATP binding cassette subfamily B member 1 (*ABCB1*), EPH receptor A7 (*EPHA7*) and RUN domain containing 3B (*RUNDC3B*) and downregulation of endothelial lipase (*LIPG*), multiple C2 and transmembrane domain containing 1(*MCTP1*), high mobility group nucleosome binding domain 5 (*HMGN5*), protocadherin 9 (*PCDH9*), protein tyrosine phosphatase receptor type K (*PTPRK*) and semaphorin 3A (*SEMA3A*) in paclitaxel-resistant cell lines. Yi Hu et al. [11] found that high stratifin (*SFN*) expression is associated with significantly worse overall survival in patients receiving gemcitabine, Taxol, Taxol combined with a platinum agent, paclitaxel or Avastin chemotherapy. In addition, Reto S Kohler et al. [12] reported that elevated maternal embryonic leucine zipper kinase(*MELK*) expression was correlated with poor survival and Taxol resistance in ovarian cancer. However, there is still a lack of research on Taxol resistance in ovarian cancer using bioinformatics methods.

In this study, we used the Gene Expression Omnibus (GEO) database to define differentially expressed genes in Taxol-sensitive and Taxol-resistant ovarian cancer cell lines. The Cancer Genome Atlas (TCGA) and GEO databases were used to determine the influence of selected genes on patient progression-free interval (PFI) and overall survival (OS). Our analysis revealed that high expression of *RIPK2* indicated poor PFI and OS. Further analysis of the mechanisms of the relationship between Taxol resistance and high expression of *RIPK2* was performed using functional analysis, pathway analysis, protein-protein interaction network and cBio Cancer Genomics Portal (cBioPortal) online tools. Overall, our study suggested that *RIPK2* could act as a biomarker for Taxol treatment sensitivity in serous ovarian cancer and provides new insights into the mechanisms underlying Taxol resistance in serous ovarian cancer.

## Methods and materials

### Identification of DEGs

The GEO datasets GSE58878, GSE26465, GSE73935 and GSE54772 were downloaded using the R package “GEOquery” [13]. The R package “limma” was used to identify DEGs in each dataset, and a heatmap was drawn using the “pheatmap” package [14]. Differences with *p* < 0.05 and |log_2_FC| > 1 were considered statistically significant. The intersections of DEGs from different datasets were determined using a Venn diagram by the R package “VennDiagram” [15].

### Survival analysis

The ovarian cancer gene expression profiles of frozen ovarian cancer tissue samples from 3 cohorts from GEO and 1 cohort from TCGA-OV were selected for survival analysis. Patients selected for our analysis were diagnosed with serous ovarian cancer and received Taxol chemotherapy, and their clinical features including OS and PFI were available. Survival analysis and two-tailed log-rank tests were carried out to compare outcomes between groups with high and low expression of certain genes using the R package “survival” [16]. Survival curves were generated by R package “survminer” [17]. The cutoff values for categorizing patients into the high and low expression groups were calculated with the maximally selected rank statistics method by the R package “survminer”. The study characteristics of the selected cohorts are described in Table 2.

### Correlation analysis

The correlation of gene expression and Taxol resistance was analyzed using the Cancer Cell Line Encyclopedia (CCLE) database which contains ovarian cancer cell line gene expression data as well as Taxol IC50 values [18]. Correlations with gene expression were analyzed in each dataset, including TCGA-OV, GSE30161, GSE32063 and GSE63885. Spearman correlation analysis was employed with the R package “corrplot” [19]. Correlation coefficients with *p* < 0.05 were considered to be statistically significant.

### Functional pathway enrichment analysis

The Gene Ontology (GO) consortium can be used to determine the related biological process (BP), cellular component (CC) and molecular function (MF) terms of a gene list. KEGG (Kyoto Encyclopedia of Genes and Genomes) is a database that integrates genomic, chemical and systemic functional information. To understand the function of *RIPK2* coexpressed genes, we applied GO and KEGG analyses with the R package “clusterProfiler” [20]. The bubble plot of top significant pathways based on the *P* value was drawn using the R package “ggplot2” [21]. *p* < 0.05 was set as the cut-off criterion.

### PPI network

A protein-protein interaction network (PPI) was used to describe interactions between proteins, providing a deep understanding of cell physiology. We generated a PPI network using the online tool STRING [22]. The obtained PPI interactions were visualized by Cytoscape (version 3.4.0, http://www.cytoscape.org/) [23].

### Evaluation of immune infiltration

The infiltration of 67 types of immune cells in ovarian cancer samples was evaluated by the R package “xCell” using the ssGSEA method [24]. The correlation between immune infiltration and *RIPK2* expression was assessed by Spearman analysis and differences with *p* < 0.05 were considered significant. A barplot was generated with the “ggplot2” package to visualize the correlation coefficients and *P* values.

### Genetic alteration analysis

cBioPortal (http://cbioportal.org) contains multiple cancer genomics datasets, including mutation, copy number variation (CNV), and gene co-occurrence information [25]. The IDs of patients who were treated with Taxol in the TCGA-OV dataset were imported into the online cBioPortal tool and *RIPK2* alterations were analyzed and visualized. The OncoPrint tab was employed to display an overview of genetic alterations of *RIPK2* per sample. The alterations and mutations of genes coexpressed with *RIPK2* were visualized with boxplots generated by cBioportal. Differences with *p* < 0.05 were considered to be statistically significant.

## Results

### Identification of DEGs using GEO datasets

**Fig. 1:**
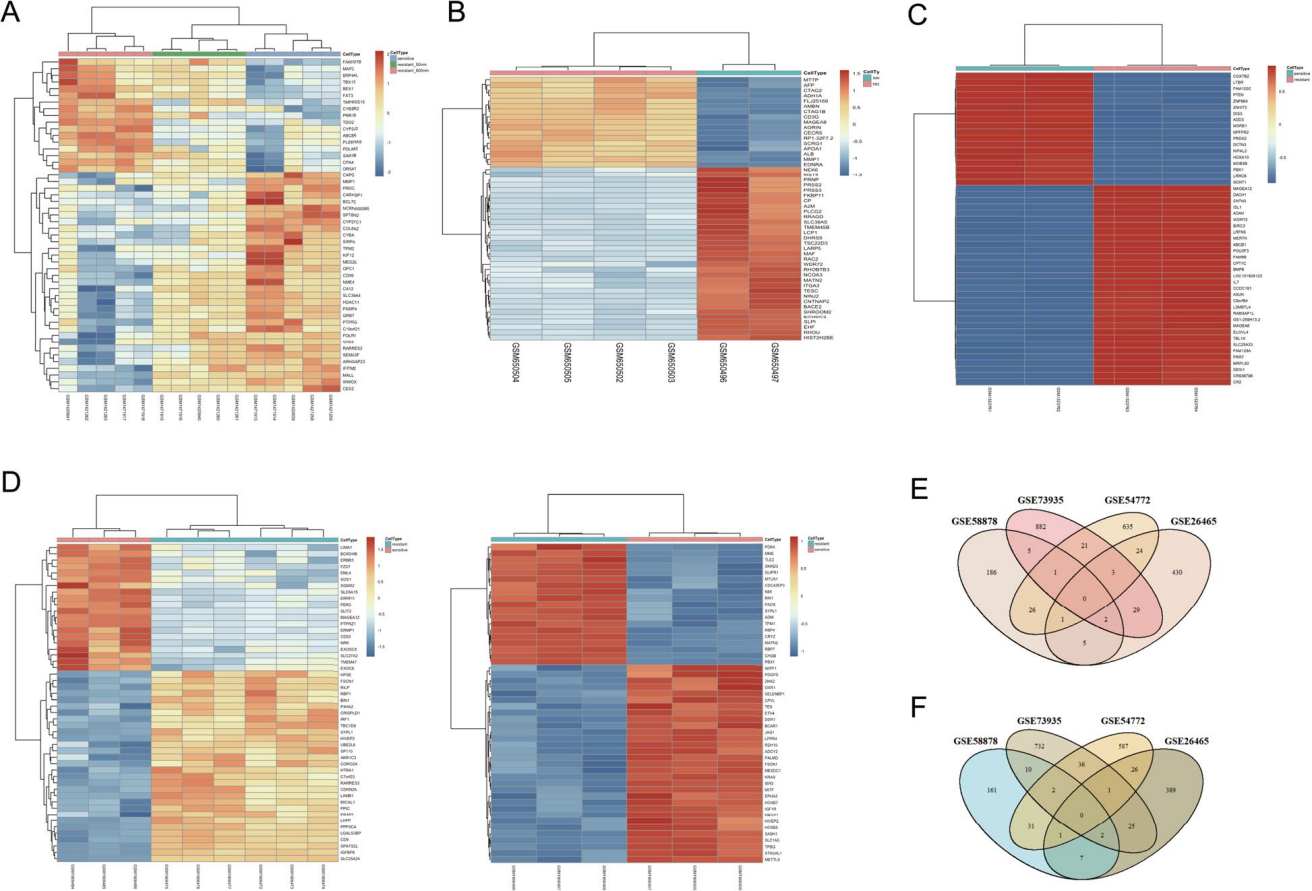
Venn diagram and heatmaps for differentially expressed genes (DEGs) in mRNA sequencing datasets. **A** heatmaps for DEGs in dataset GSE58878. **B** heatmaps for DEGs in dataset GSE26465. **C** heatmaps for DEGs in dataset GSE73935. **D** heatmaps for DEGs in dataset GSE54772. **E** Venn diagram showing the intersection of the upregulated DEGs from datasets GSE58878, GSE26465, GSE73935 and GSE54772. **F** Venn diagram showing the intersection of the downregulated DEGs from datasets GSE58878, GSE26465, GSE73935 and GSE54772

The GEO datasets GSE58878, GSE26465, GSE73935 and GSE54772, containing the expression profiles of Taxol-sensitive and Taxol-resistant cell lines, were downloaded using the R package “GEOquery”. The study characteristics and sizes of the selected datasets are described in Table 1. A total of 226 upregulated genes and 214 downregulated genes were found in Taxol-resistant SKOV3 cells in GSE58878 microarray data (Fig. 1A and Supplementary Table 1), while 494 upregulated genes and 451 downregulated genes were identified in Taxol-resistant OV90 cell line in GSE26465 (Fig. 1B and Supplementary Table 1). A total of 150 DEGs were identified from the GSE73935 dataset, including 71 upregulated genes and 79 downregulated genes in the Taxol-resistant A2780 and W1 cell line (Fig. 1C and Supplementary Table 1). Additionally, 74 and 48 genes were up-regulated and down-regulated respectively in Taxol-resistant SKOV3 cell line in the GSE54772 dataset (Fig. 1D and Supplementary Table 1). The overlapping upregulated and downregulated genes were obtained from the intersection of the DEG datasets identified above (Fig. 1E, F).

**Table 1:** mRNA sequencing datasets containing Taxol-sensitive/resistant cell lines

Accession number of dataset	Platform	Cell line	Response to Taxol	
			Sensitive	Resistant
GSE58878	GPL16951	SKOV3	5	10
GSE26465	GPL6104	OV90	2	4
GSE73935	GPL13667	A2780	3	6
		W1	3	3
GSE54772	GPL570	SKOV3	2	2

### Survival analysis

To explore whether the DEGs identified in Taxol-sensitive and Taxol-resistant ovarian cancer cell lines are related to the PFI and OS of ovarian cancer patients, samples from TCGA-OV with recurrence and therapy information were selected and analyzed (Supplementary Table 2). For each DEG identified, the correlation of the expression of this DEG with PFI and OS was evaluated with the Kaplan-Meier method. Samples were divided into a high-expression group and a low-expression group according to the cutoff value for the specific DEG, which was calculated by the maximally selected rank statistics method using the R package “survminer”. For genes that showed statistically significant differences in the OS and PFI survival analysis, we determined whether the difference in their expression in sensitive and drug-resistant cell lines was consistent with the differences shown in the survival analysis. If a specific gene had a higher expression level in the Taxol-resistant cell line, the survival of patients with high expression of this gene should be poorer. Interferon stimulated gene 15 (*ISG15*), synuclein alpha (*SNCA*) and *RIPK2* were upregulated in Taxol-resistant cell lines, and their high expression was also correlated with shorter OS and PFI in the TCGA-OV dataset. Phospholipase C gamma 2 (*PLCG2*), ras homolog family member U (*RHOU*), tribbles pseudokinase 2 (*TRIB2*) and elongator acetyltransferase complex subunit 3 (*ELP3*) had low expression in Taxol-resistant cell lines and their high expression was related to better survival in the TCGA-OV dataset (Supplementary Figures S1, S2, S3, S4, S5, S6).

**Fig. 2:**
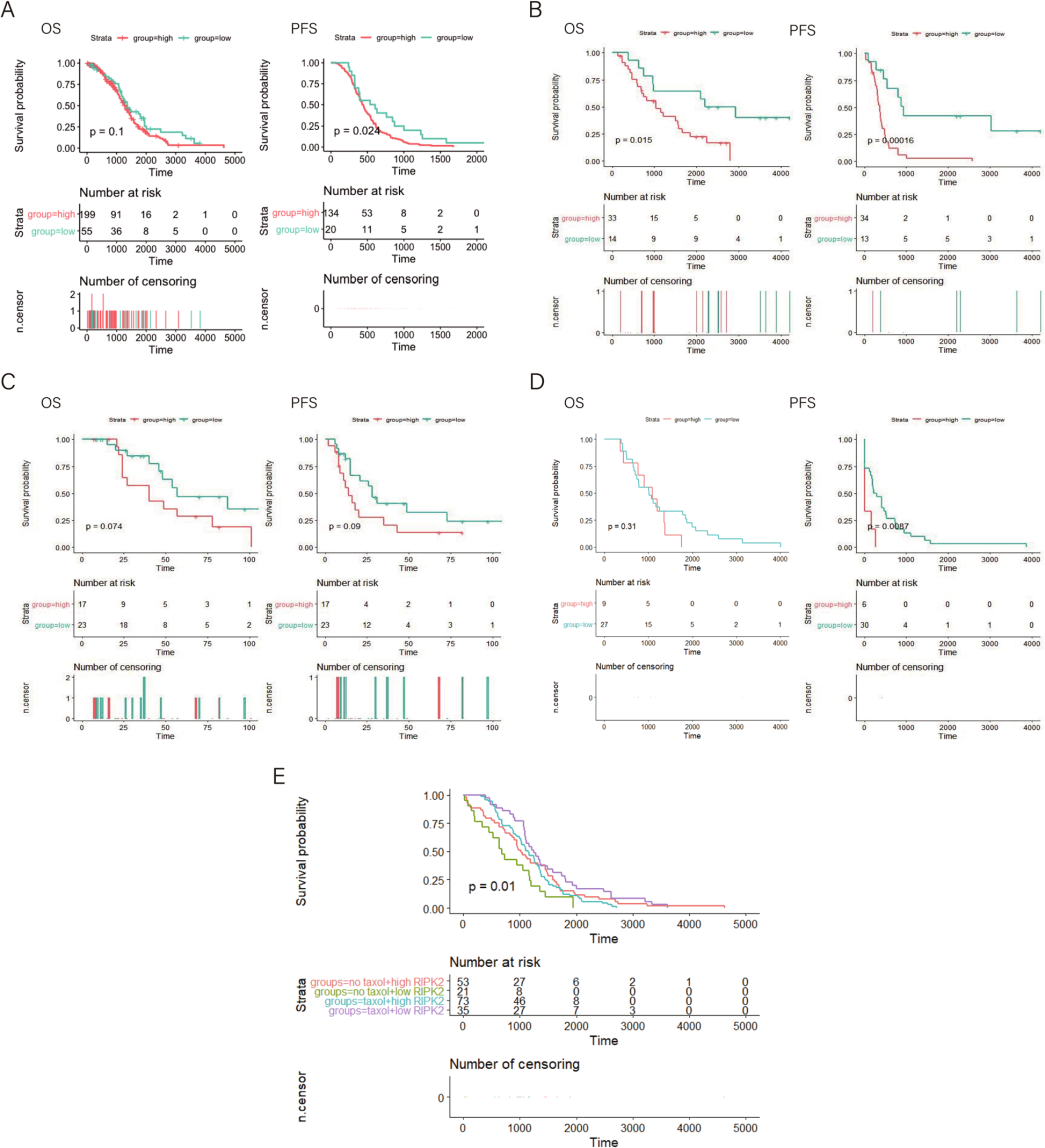
Relationship of *RIPK2* expression with survival outcome. **A** Overall survival (OS) and progression-free interval (PFI) in *RIPK2* high and low expression groups in the TCGA-OV dataset. **B** Overall survival (OS) and progression-free interval (PFI) in the *RIPK2* high and low expression groups in the GSE30161 dataset. **C** Overall survival (OS) and progression-free interval (PFI) in the *RIPK2* high and low expression groups in the GSE32063 dataset. **D** Overall survival (OS) and progression-free interval (PFI) in the *RIPK2* high and low expression group in the GSE63885 dataset. **E** Overall survival (OS) of groups defined by *RIPK2* expression and Taxol usage in the TCGA-OV cohort. The numbers below the figures denote the number of patients at risk in each group

Datasets GSE30161, GSE32062 and GSE63885, which contain clinical information of patients with serous ovarian cancer, including OS and PFI data, were selected to further validate the effects of the expression of *ISG15*, *SNCA*, *RIPK2*, *PLCG2*, *RHOU*, *TRIB2* and *ELP2* on patient sensitivity to Taxol treatment (Table 2 and Supplementary Figures 1, 2, 3, 4, 5, 6). In all three datasets, the *RIPK2* high expression group and *RIPK2* low expression group showed significant differences in survival in terms of OS and PFI, suggesting that high expression of *RIPK2* is a risk factor for survival in patients with serous ovarian cancer (Fig. 2).

**Table 2:** mRNA sequencing datasets containing overall survival and progress free interval of serous ovarian patients treated with Taxol

Accession number of dataset	Platform	Pathological type	Samples treated with Taxol
GSE30161	GPL570	serous cancer (85%)	58
GSE32063	GPL6480	advanced-stage high-grade serous ovarian cancer	40
GSE63885	GPL570	serous cancer	36

We further validated the predictive value of *RIPK2* expression by dividing the TCGA-OV cohort into four groups based on patient *RIPK2* expression levels and whether Taxol was used during treatment. Survival analysis was carried out and we found that patients with low *RIPK2* expression and Taxol treatment showed the longest OS, while those who had low *RIPK2* expression but were not treated with Taxol showed the shortest OS. Furthermore, there was no significant difference in OS between patients treated with Taxol and those not treated with Taxol in the group of patients with high expression of *RIPK2*.

### Correlations of *RIPK2* gene expression with Taxol resistance in CCLE

CCLE contains a large panel of human cancer cell lines and their pharmacological profiles, including the gene expression profiles and IC50 values to Taxol of 21 ovarian cancer cell lines (Supplementary Table 3). By dividing the expression level of *RIPK2* in each cell line by the expression level of *GAPDH* in the same cell line, the expression of *RIPK2* was normalized. The correlation between *RIPK2* expression and IC50 value of Taxol was analyzed by the R package “corrplot” with the Spearman method. The correlation coefficient was 0.46 (*p* < 0.05), indicating that higher expression of *RIPK2* was associated with Taxol resistance of multiple ovarian cancer cell lines.

### *RIPK2* coexpression network in ovarian cancer

To gain further insight into the biological function of *RIPK2* in the development of Taxol resistance in ovarian cancer, the coexpressed genes of *RIPK2* in serous ovarian cancer patients treated with Taxol were analyzed. In the TCGA-OV dataset, 341 genes were found to show a significant positive coexpression pattern with *RIPK2*, while no gene showed negative coexpression pattern. The expression of 704 genes had a positive correlation with *RIPK2* expression in GSE30161 and 1706 had a negative correlation. 45 genes were positively coexpressed while 37 were negatively coexpressed with *RIPK2* in GSE32063. 17 genes were found to have a positive coexpression pattern with *RIPK2* in dataset GSE63885 and 4 genes had a negative coexpression relationship. A description of the coexpressed genes is detailed in Supplementary Table 4.

**Fig. 3:**
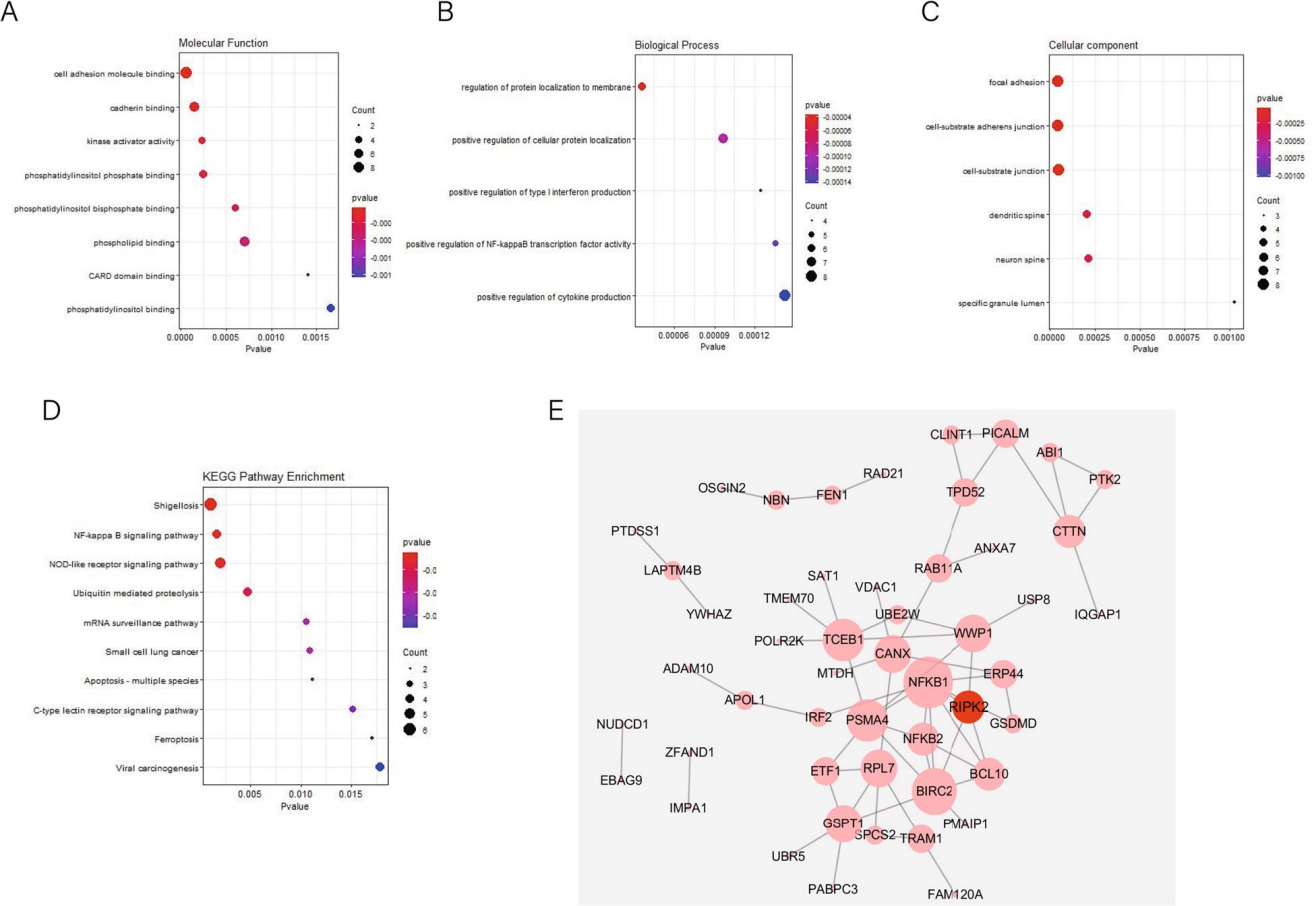
Gene Ontology (GO) and Kyoto Encyclopedia of Genes and Genomes(KEGG) enrichment analyses and protein-protein network(PPI) analysis of coexpressed genes of *RIPK2* in serous ovarian cancer patients treated with Taxol. **A** Molecular function. **B** Biological process. **C** cellular component. **D** Enriched KEGG pathways of genes coexpressed with RIPK2. The horizontal axis represents the number of DEGs under the GO/KEGG term and the sizes of the dots represents the number of genes located in the functional area. **E** PPI network generated by the STRING database and visualized by Cytoscape. Nodes represent coexpressed genes and edges represent PPIs

Functional analysis were performed using the intersections of coexpressed genes in every two datasets as input. Significant GO terms showed that *RIPK2* coexpressed genes from multiple datasets mainly participated in cell adhesion molecule binding, positive regulation of cytokine production and focal adhesion (Fig. 3A-C). KEGG pathway analysis showed enrichment in the NF-kappa B signaling pathway, NOD-like receptor signaling pathway and ubiquitin mediated proteolysis pathway (Fig. 3D and Supplementary Table 5).

A PPI network of *RIPK2* related genes was created on the basis of information from the STRING database, which further illustrated the connection of the coexpressed genes at the protein level. The average aggregation coefficient was 0.508, and the enrichment *p* value was less than 0.001 (Fig. 3E).

### Genomic alterations of *RIPK2* in ovarian cancer

**Fig. 4:**
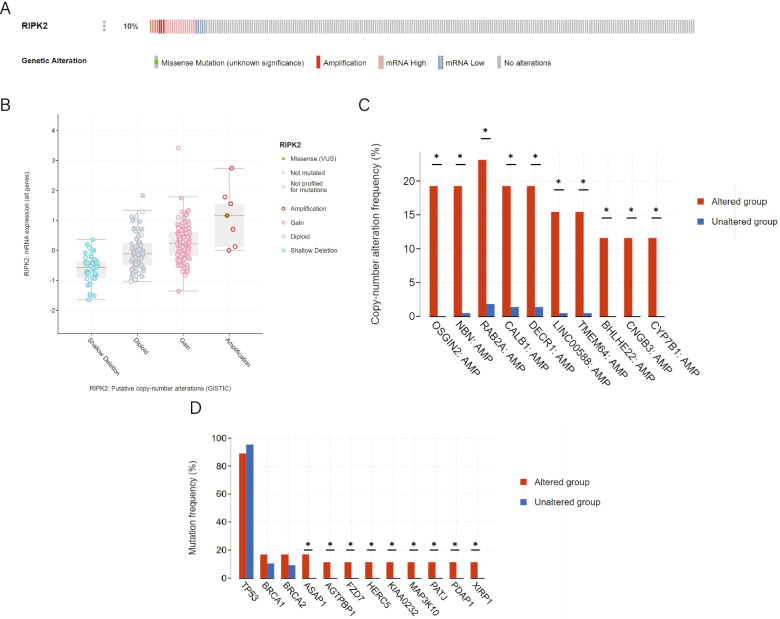
*RIPK2* genomic alterations in ovarian cancer (cBioPortal). **A** OncoPrint of *RIPK2* alterations in TCGA-OV cohort. Different types of genetic alterations are highlighted in different colors. **B** the relationship of copy number alterations and mRNA expression of *RIPK2*. **C** difference of genetic mutations in *RIPK2* altered and unaltered group. Tumor protein p53 (*TP53*), BReast CAncer gene 1 (*BRCA1*), BReast CAncer gene 2 (*BRCA2*) and 10 other genes with the most significant *p* values were shown. **D** copy-number change. 10 genes with the most significant p values were shown. *p < 0.01

The cBioPortal tool was used to determine the alterations in *RIPK2* in ovarian cancer patients who were treated with Taxol in the TCGA-OV database. Alterations occurred in 26 of 252 samples (10%), including 1 missense mutation (0.4%), 7 amplifications (3%), 21 cases of mRNA upregulation (8%) and 4 cases of mRNA downregulation (2%) (Fig. 4A). *RIPK2* amplification results in high expression of *RIPK2*, which may be related to Taxol resistance. AMP was the most common type of *RIPK2* copy number alteration (CNA) in ovarian cancer (Fig. 4B). Furthermore, there was a significant difference in the amplification of oxidative stress induced growth inhibitor family member 2 (*OSGIN2*), nibrin (*NBN*), Ras-related protein Rab-2A (*RAB2A*) and calbindin 1 (*CALB1*) in the *RIPK2*-altered and *RIPK2*-unaltered groups (Fig. 4C and Supplementary Table 6). Moreover, the mutation frequency of ArfGAP with SH3 domain, ankyrin repeat and PH domain 1(*ASAP1*), ATP/GTP binding protein 1(*AGTPBP1*), frizzled class receptor 7(*FZD7*), HECT and RLD domain containing E3 ubiquitin protein ligase 5 (*HERC5*), *KIAA0232*, mitogen-activated protein kinase kinase kinase 10 (*MAP3K10*), PATJ crumbs cell polarity complex component (*PATJ*), PDGFA associated protein 1(*PDAP1*), and xin actin binding repeat containing 1(*XIRP1*) was significantly associated with the alteration of *RIPK2* (Fig. 4D and Supplementary Table 7).

### Evaluation of the difference in immune cell infiltration

**Fig. 5:**
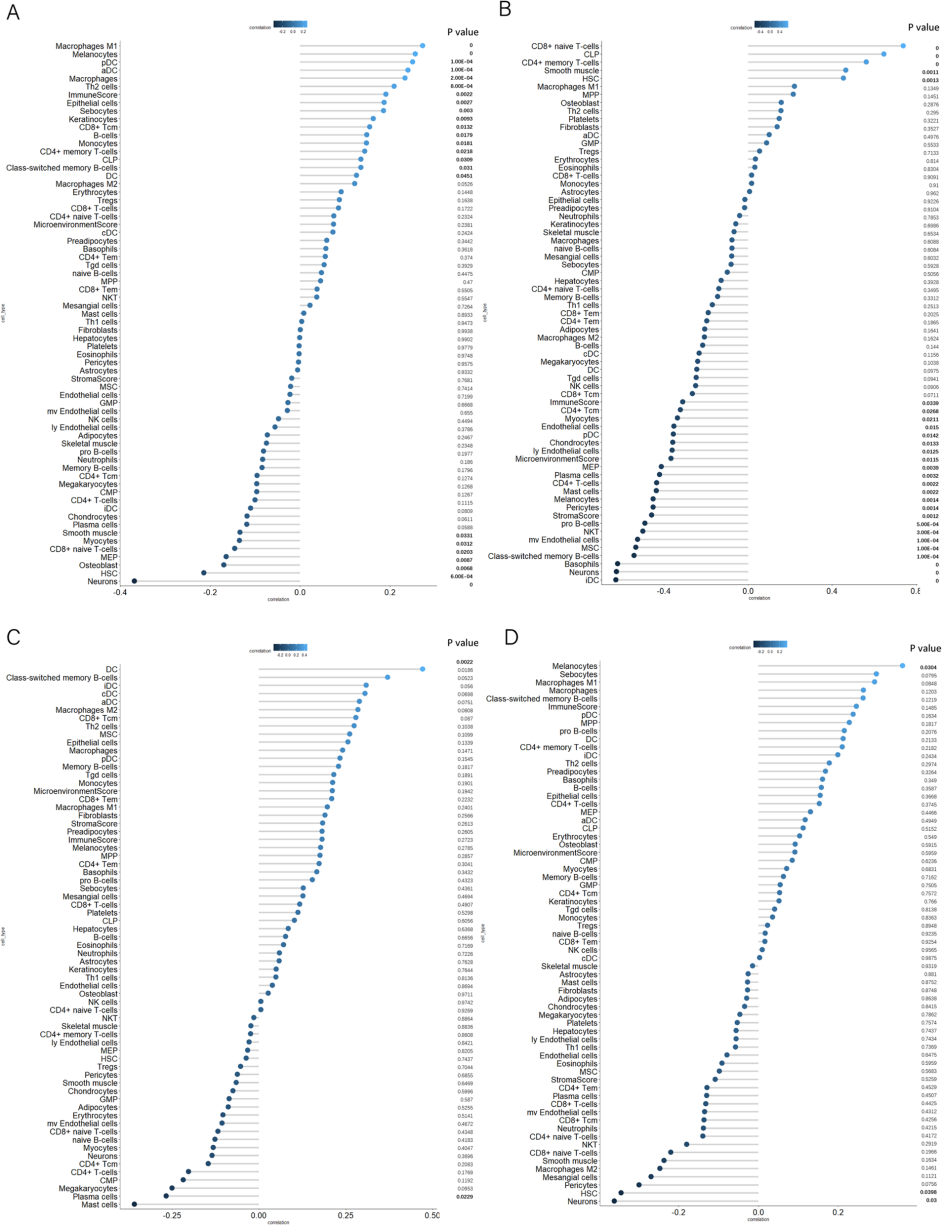
Correlation between *RIPK2* expression and immune infiltration **A** Correlation between *RIPK2* expression and infiltrating immune cells in TCGA-OV dataset. **B** Correlation between *RIPK2* expression and infiltrating immune cells in GSE30161 dataset. **C** Correlation between *RIPK2* expression and infiltrating immune cells in GSE32063 dataset. **D** Correlation between *RIPK2* expression and infiltrating immune cells in GSE63885 dataset. *p* < 0.05 was considered statistically significant. MPP, Multipotent rogenitors; CD8+ Tem, CD8+ effector memory T-cells; CMP, Common myeloid progenitors; GMP, Granulocyte-macrophage progenitors; MEP, Megakaryocyte–erythroid progenitors; Tregs, Regulatory T-cells; HSC, Hematopoietic stem cells; CD4+ Tcm, CD4+ central memory T-cells; mv Endothelial cells, Microvascular endothelial cells; CD4+ Tem, CD4+ effector memory T-cells; CD8+ Tcm, CD8+ central memory T-cells; ly Endothelial cells, Lymphatic endothelial cells; MSC, Mesenchymal stem cells; aDC, Activated dendritic cells; cDC, Xonventional dendritic cells; pDC, Plasmacytoid dendritic cells; iDC, Immature dendritic cells; Th2 cells, Type 2 T-helper cells; CLP, Common lymphoid progenitors; Th1 cells, Type 1 T-helper cells; NKT, Natural killer T-cells; Tgd cells, Gamma delta T-cells

The immune infiltration of 64 types of immune cells, including adaptive and innate immune cells, hematopoietic progenitors, epithelial cells, and extracellular matrix cells, were evaluated by the R package “xCell”, using the ssGSEA method in ovarian cancer tissues. In the TCGA-OV dataset, the infiltration of M1 macrophages, melanocytes and plasmacytoid dendritic cells (pDCs) was positively related to the expression of *RIPK2*, while the infiltration of neurons was negatively related to the expression of *RIPK2* (Fig. 5A and Supplementary Tables 8 and 9). Furthermore, CD8+ naive T-cells, common lymphoid progenitors (CLPs), CD4+ memory T cells, smooth muscle cells and hematopoietic stem cells (HSCs) showed increased infiltration when *RIPK2* expression levels were higher, but immature dendritic cells (iDCs), neurons, basophils, class-switched memory B cells, mesenchymal stem cells (MSCs), microvascular endothelial cells, natural killer T cells (NKTs), pro-B cells, pericytes, melanocytes, mast cells, CD4+ T cells, plasma cells, MEPs, lymphatic endothelial cells, chondrocytes, pDCs, endothelial cells, myocytes and CD4+ central memory T cells showed decreased infiltration (Fig. 5B and Supplementary Tables 8 and 9). The infiltration of dendritic cells (DCs) was positively correlated with the expression of *RIPK2* and the infiltration of mast cells was negatively correlated with the expression of *RIPK2* in dataset the GSE32063 dataset (Fig. 5C and Supplementary Tables 8 and 9). In the GSE63885 dataset, melanocyte infiltration was high when *RIPK2* expression was higher, while the infiltration of neurons and HSCs was low (Fig. 5D and Supplementary Tables 8 and 9).

## Discussion

Serous ovarian cancer, a type of epithelial ovarian cancer, is conventionally treated by cytoreductive surgery and chemotherapy based on platinum agents and Taxol [26]. However, many patients die because of the development of chemoresistance during platinum and Taxol treatment. While platinum resistance has gained more attention in ovarian cancer studies, a detailed understanding of potential biomarkers associated with Taxol-resistance in ovarian cancer treatment is still lacking. In this study, bioinformatic methods were used, and a total of 103 common DEGs (54 upregulated and 49 downregulated) were found in 4 GEO datasets of Taxol-sensitive and Taxol-resistant cell lines. *ISG15*, *SNCA*, *RIPK2*, *PLCG2*, *RHOU*, *TRIB2* and *ELP2* influenced the OS and PFI of ovarian cancer patients in the TCGA-OV dataset, while *RIPK2* also affected the OS and PFI of ovarian cancer patients treated with Taxol in the GSE30161, GSE32062 and GSE63885 datasets. Thus, that higher expression of *RIPK2* may lead to Taxol resistance in serous ovarian cancer was validated via combined DEG analysis and survival analysis. The reliability of *RIPK2* as a marker of Taxol resistance was further verified when we divided the TCGA-OV cohort into four groups based on Taxol treatment and *RIPK2* expression. The OS of patients who had high expression of *RIPK2* and were treated with Taxol was significantly shorter than that of patients with lower expression of *RIPK2*, which might suggest that patients with high expression of *RIPK2* were tend to be sensitive to Taxol treatment. This idea was confirmed when we found that the expression of *RIPK2* was positively related to the IC50 of Taxol in multiple ovarian cancer cell lines using data from the CCLE database.

*RIPK2* belongs to the receptor-interacting protein (RIP) kinase family and serves as a key molecule regulating inflammatory signaling and cell-death pathways [27]. *RIPK2* mediated signaling responses are initiated by the bacteria-sensing pattern recognition receptors nucleotide-binding oligomerization domain-containing proteins 1 and 2 (NOD1/2). Previous studies have shown that *RIPK2* might be responsible for the chronic inflammation of inflammatory bowel disease (IBD) [28, 29], and the high level of *RIPK2* expression was associated with advanced tumors and metastasis of inflammatory breast cancer [30]. It has also been reported that *RIPK2* polymorphisms are related to tumor infiltration degree, lymph node metastasis and survival in urothelial bladder cancer [31] and susceptibility to gastric cancer [32].

To assess the roles of *RIPK2* in Taxol resistance in serous ovarian cancer, we identified its coexpressed genes in samples from patients treated with Taxol from the TCGA-OV dataset. The coexpressed genes were mainly enriched in the biological process terms regulation of protein localization to membrane and the positive regulation of cellular protein localization, type I interferon production, nuclear factor kappa-light-chain-enhancer of activated B cells (NF-κB) transcription factor activity and cytokine production. The PPI network analysis showed that *RIPK2* was positively coexpressed with nuclear factor kappa B subunit 1 (*NFKB1*), baculoviral IAP repeat containing 2 (*BIRC2*), etc. The pathway analysis of *RIPK2* coexpressed genes also demonstrated that these genes took part in the positive regulation of NF-κB transcription factor activity. Although previous studies revealed that the effectiveness of Taxol to ovarian cancer can be regulated by multiple pathways, including cell death related pathways, such as the JNK/SAPK pathway, the p53 pathway [33] and signaling pathways like the PI3K/AKT pathway [34, 35], the FAK/Rho pathway [36] etc., pathway analysis of *RIPK2* and its coexpressed genes didn’t show enrichment in these pathways, which might suggest that *RIPK2* participates in Taxol-resistant ovarian cancer by activating NF-κB mediated transcription [37].

Recently, cancer-associated gene alterations have been studied in pan-cancer databases, revealing that CNA might be a marker of somatic genomic mutations in cancer genome that lead to tumorigenesis. Oncogenic driver genes with increased copy number and expression can be used as potential drug targets for tumor targeted therapy [38]. In our study, *RIPK2* alterations were found in 10% of ovarian cancer patients who were treated with Taxol. Furthermore, mRNA upregulation occurred most frequently and the major type of genomic alteration was amplification. Such amplification resulted in high expression of *RIPK2* compared with that seen in the diploid, gain or shallow depletion group. We also found that the copy number of *OSGIN2*, and *NBN* differed in *RIPK2*-altered and *RIPK2*-unaltered patients, and these genes were coexpressed with *RIPK2* in ovarian cancer. Rohit Mehra et al. [39] found that *RIPK2-OSGIN2* gene fusion could occur in patients suffering from primary clear-cell adenocarcinoma of the urethra. However, there have been no reports on *RIPK2* related gene alterations in ovarian cancer. The mutation frequency of 10 genes, including *ASAP1*, *AGTPBP1* and *FZD7* etc., differed when *RIPK2* expression differed. ASAP1 and RIPK2 were reported as hub proteins of inflammatory bowel disease and colorectal cancer; and *ASAP1* expression might be associated with pulmonary and bladder neoplasms [40]. Therefore, our research suggests that *ASAP1* mutation might be related to *RIPK2* alteration and thus be associated with Taxol resistance in ovarian cancer.

Immune infiltration is reported to have a tight association with tumor progression and prognosis, and could be a markers for drug response in multiple tumors [41]. Ellen L Goode et al. [42] has reported that CD8+ T lymphocyte infiltration was significantly associated with longer overall survival in HGSOCs. In this study, the ssGSEA method was applied by using the R package “xCell”, and the correlation of immune cell infiltrations with *RIPK2* was analyzed. The infiltration of neurons was found to be negatively correlated with *RIPK2* expression in three datasets, and MEPs and mast cells were found to have negative correlations with RIPK2 in two datasets. The infiltration of DCs, CD4+ memory T cells and CLPs was positively correlated with *RIPK2* expression in two datasets. These results suggest that high expression of *RIPK2* can influence the tumor microenvironment by affecting the infiltration of neurons, DCs, CD4+ memory T cells and CLPs.

In summary, we found that high expression of *RIPK2* might be associated with the resistance of Taxol in serous ovarian cancer by identifying common DEGs and performing survival analysis with multiple datasets. Our results suggest that *RIPK2* upregulation is likely to cause resistance to Taxol by controlling the infiltration of immune cells. The expression of *RIPK2* was significantly correlated with the expression of *NFKB1*, indicating that Taxol resistance might be related to the activation of NOD1/RIPK2/NF-κB inflammatory pathways. However, further experimental validation is required to confirm of these results. These findings provide novel insights into the use of *RIPK2* as a biomarker for Taxol resistance and its possible mechanisms, paving the way for a possible solution to Taxol resistance in serous ovarian cancer.

## Supplementary Information

**Additional file 1: Supplementary Table 1.** DEGs_of_4_datasets.

**Additional file 2: Supplementary Table 2.** IDs_of_samples_with_taxol_treatment.

**Additional file 3: Supplementary Table 3.** Taxol_IC50_and_RIPK2_expression.

**Additional file 4: Supplementary Table 4.** Genes_co-expressed_with_RIPK2.

**Additional file 5: Supplementary Table 5.** GO,KEGG_and_PPI_analysis.

**Additional file 6: Supplementary Table 6.** Relationship_of_CNA_with_RIPK2.

**Additional file 7: Supplementary Table 7.** Relationship_of_mutation_with_RIPK2.

**Additional file 8: Supplementary Table 8.** Immune_infiltration.

**Additional file 9: Supplementary Table 9.** RIPK2_expression_immune_infiltration.

**Additional file 10: Supplementary Figure 1-6.** Relationship of expression of *ISG15*, *SNCA*, *PLCG2*, *RHOU*, *TRIB2* with survival outcome of serous ovarian cancer.

## Data Availability

The datasets generated and/or analyzed in this study are available in the TCGA repository (https://portal.gdc.cancer.gov/repository?facetTab=cases) and GEO repository (https://www.ncbi.nlm.nih.gov/geo/query/acc.cgi?acc=GSE58878, https://www.ncbi.nlm.nih.gov/geo/query/acc.cgi?acc=GSE26465, https://www.ncbi.nlm.nih.gov/geo/query/acc.cgi?acc=GSE73935, https://www.ncbi.nlm.nih.gov/geo/query/acc.cgi?acc=GSE54772, https://www.ncbi.nlm.nih.gov/geo/query/acc.cgi?acc=GSE30161, https://www.ncbi.nlm.nih.gov/geo/query/acc.cgi?acc=GSE32063, https://www.ncbi.nlm.nih.gov/geo/query/acc.cgi?acc=GSE63885).
