# Supplementary material for: High expression of RIPK2 is associated with Taxol resistance in serous ovarian cancer
Source: J Ovarian Res. 2022 Apr 27;15:48. doi: 10.1186/s13048-022-00986-2 (PMC9044796; doi:10.1186/s13048-022-00986-2)
Supplement: Supplementary file 10 — Additional file 10: Supplementary Figure 1-6. Relationship of expression of ISG15, SNCA, PLCG2, RHOU, TRIB2 with survival outcome of serous ovarian cancer. [file 13048_2022_986_MOESM10_ESM.docx]

**Supplementary Materials**

**High Expression of *RIPK2* is Associated with Taxol Resistance in Serous Ovarian Cancer**

Yuqing Shen^1^, Hui Lin^1^, Yihua Wu ^2,*^, Weiguo Lu^1 *^

^1^ Department of Gynecologic Oncology of Women's Hospital, Zhejiang University School of Medicine, Hangzhou, Zhejiang, 310058, China.

^2^ Department of Toxicology of School of Public Health, and Department of Gynecologic Oncology of Women's Hospital, Zhejiang University School of Medicine, Hangzhou, Zhejiang, 310058, China.

^*^Correspondence and requests for materials should be addressed to lbwg@zju.edu.cn and [georgewu@zju.edu.cn](mailto:georgewu@zju.edu.cn)

**
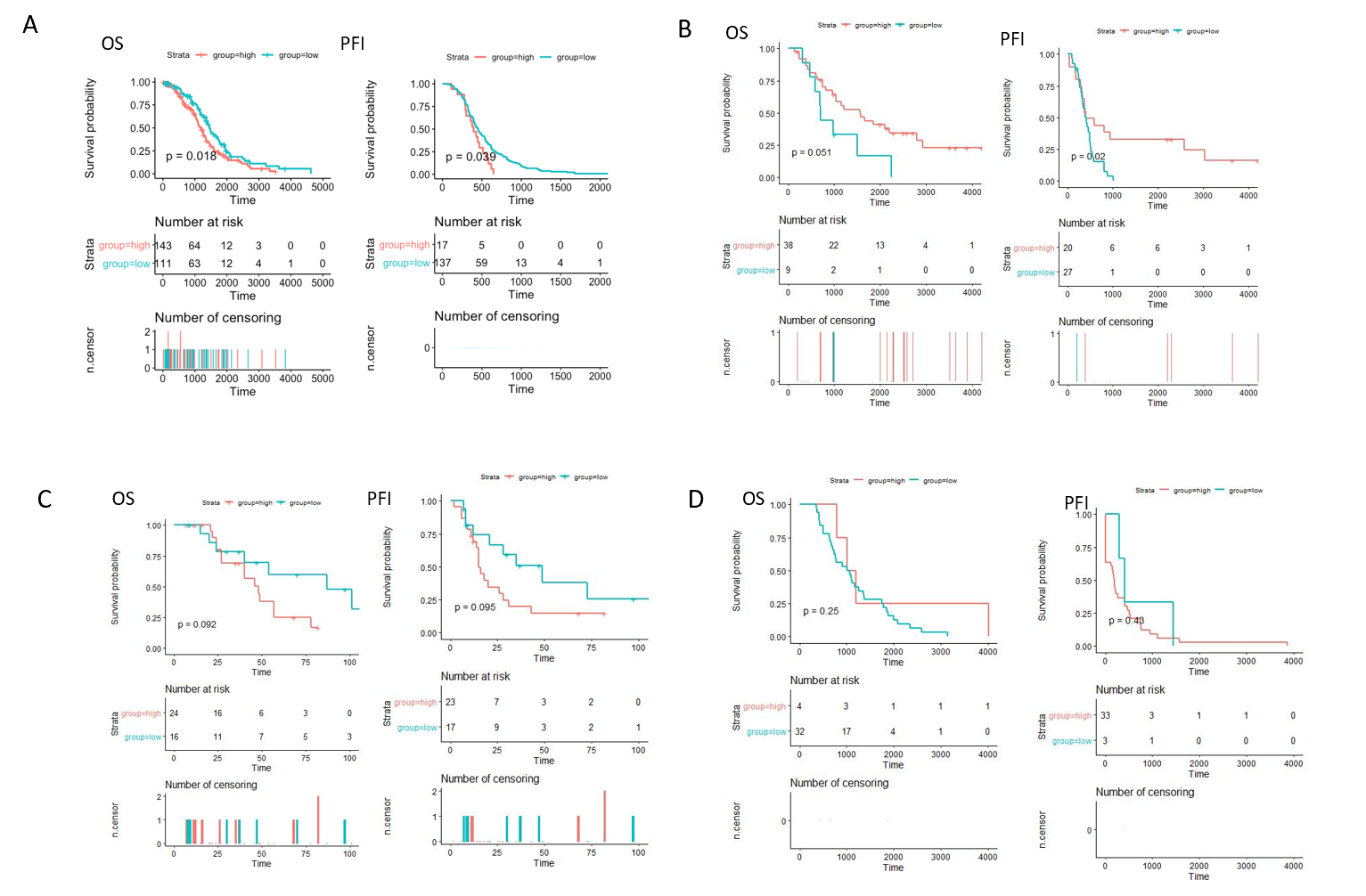
**

**Supplementary Figure 1.** Relationship of *ISG15* expression with survival outcome. (A) Overall survival (OS) and progression free interval (PFI) in the *ISG15* high and low expression groups in the TCGA-OV dataset. (B) Overall survival (OS) and progression free interval (PFI) in the *ISG15* high and low expression groups in the GSE30161 dataset. (C) Overall survival (OS) and progression free interval (PFI) in the *ISG15* high and low expression groups in the GSE32063 dataset. (D) Overall survival (OS) and progression free interval (PFI) in the *ISG15* high and low expression groups in GSE63885 dataset. The numbers below the figures denote the number of patients at risk in each group.

**
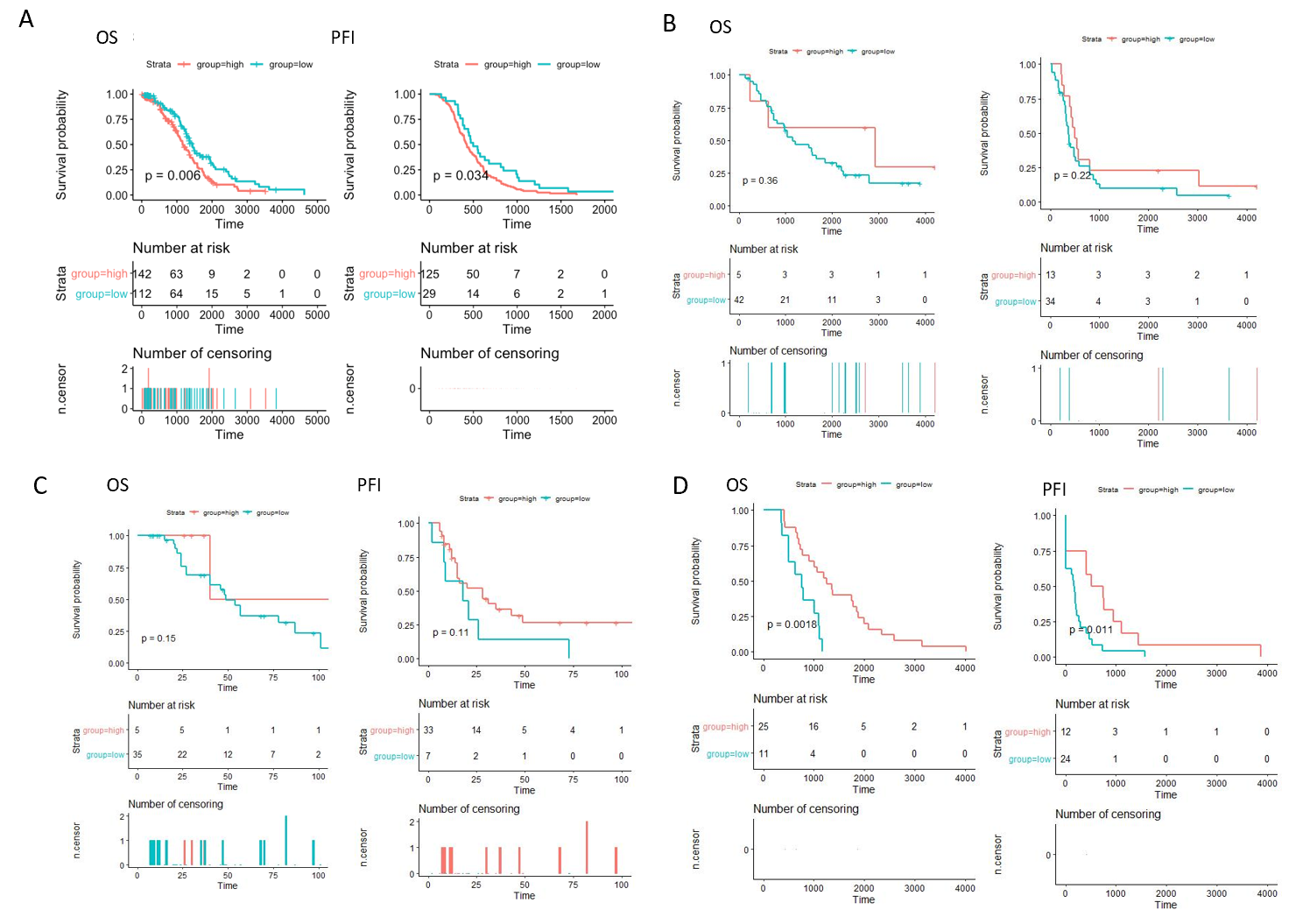
**

**Supplementary Figure 2.** Relationship of *SNCA* expression with survival outcome. (A) Overall survival (OS) and progression free interval (PFI) in the *SNCA* high and low expression groups in the TCGA-OV dataset. (B) Overall survival (OS) and progression free interval (PFI) in the *SNCA* high and low expression groups in the GSE30161 dataset. (C) Overall survival (OS) and progression free interval (PFI) in the *SNCA* high and low expression groups in the GSE32063 dataset. (D) Overall survival (OS) and progression free interval (PFI) in the *SNCA* high and low expression groups in the GSE63885 dataset. The numbers below the figures denote the number of patients at risk in each group.

**
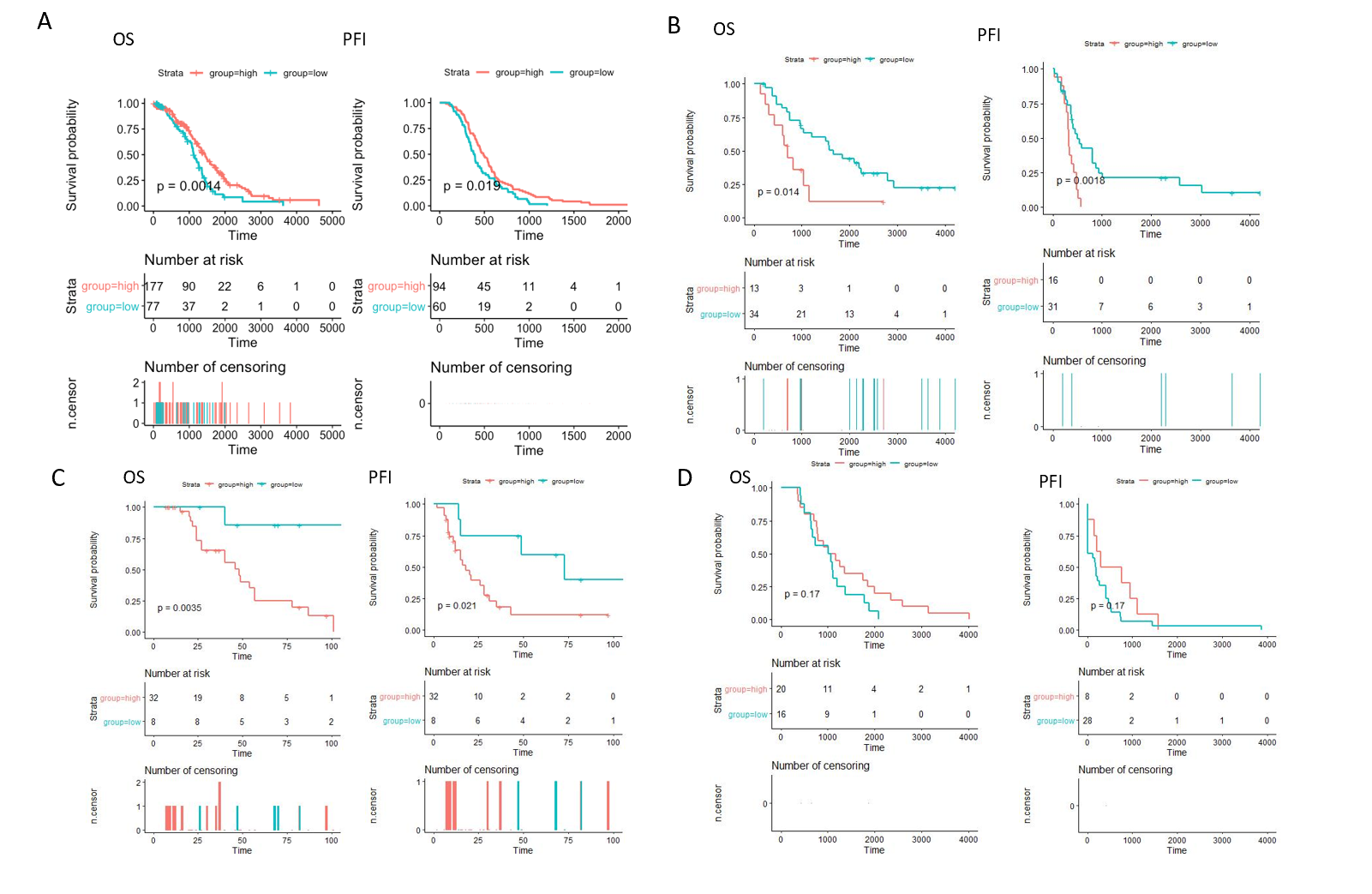
**

**Supplementary Figure 3.** Relationship of *PLCG2* expression with survival outcome. (A) Overall survival (OS) and progression free interval (PFI) in the s*PLCG2* high and low expression groups in the TCGA-OV dataset. (B) Overall survival (OS) and progression free interval (PFI) in the *PLCG2* high and low expression groups in the GSE30161 dataset. (C) Overall survival (OS) and progression free interval (PFI) in the *PLCG2* high and low expression groups in GSE32063 datasets. (D) Overall survival (OS) and progression free interval (PFI) in the *PLCG2* high and low expression groups in the GSE63885 datasets. The numbers below the figures denote the number of patients at risk in each group.

**
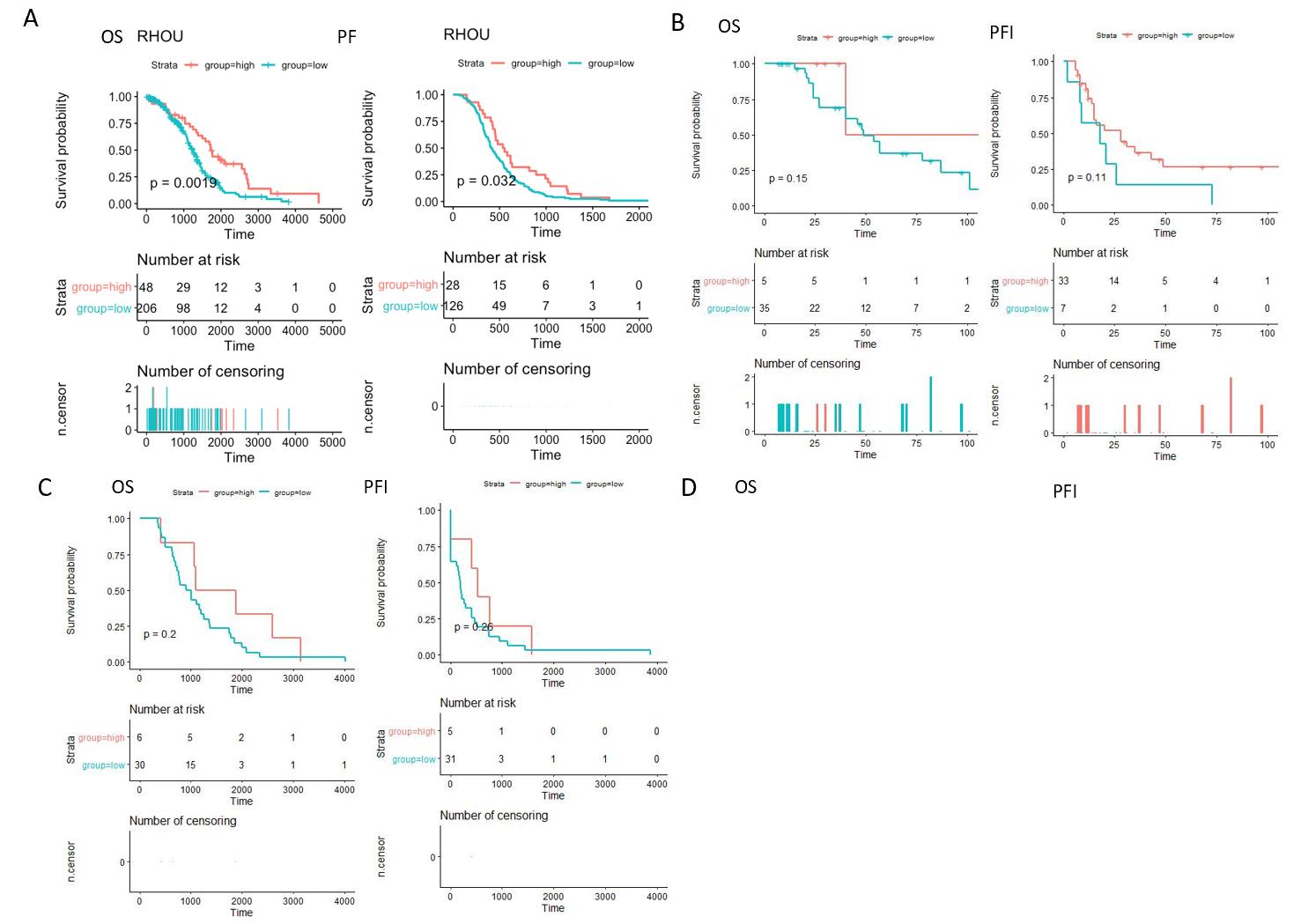
**

**Supplementary Figure 4.** Relationship of *RHOU* expression with survival outcome. (A) Overall survival (OS) and progression free interval (PFI) in the *RHOU* high and low expression groups in the TCGA-OV dataset. (B) Overall survival (OS) and progression free interval (PFI) in the *RHOU* high and low expression groups in the GSE32063 dataset. (C) Overall survival (OS) and progression free interval (PFI) in the *RHOU* high and low expression groups in the GSE63885 dataset. The numbers below the figures denote the number of patients at risk in each group.

**
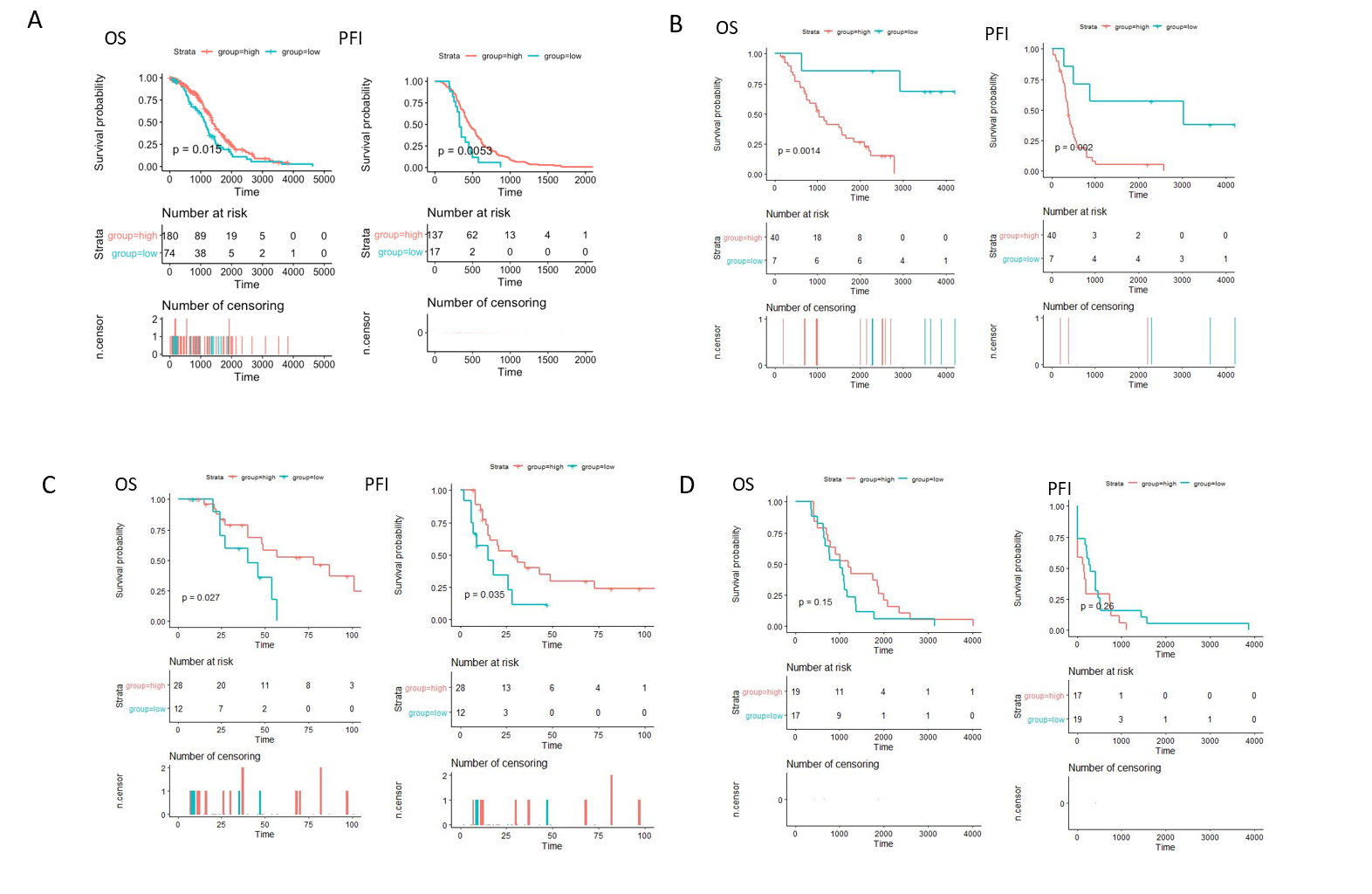
**

**Supplementary Figure 5.** Relationship of *TRIB2* expression with survival outcome. (A) Overall survival (OS) and progression free interval (PFI) in the *TRIB2* high and low expression groups in the TCGA-OV dataset. (B) Overall survival (OS) and progress free interval (PFI) in the *TRIB2* high and low expression groups in the GSE30161 dataset. (C) Overall survival (OS) and progression free interval (PFI) in the *TRIB2* high and low expression groups in the GSE32063 dataset. (D) Overall survival (OS) and progression free interval (PFI) in the *TRIB2* high and low expression groups in the GSE63885 dataset. The numbers below the figures denote the number of patients at risk in each group.

**
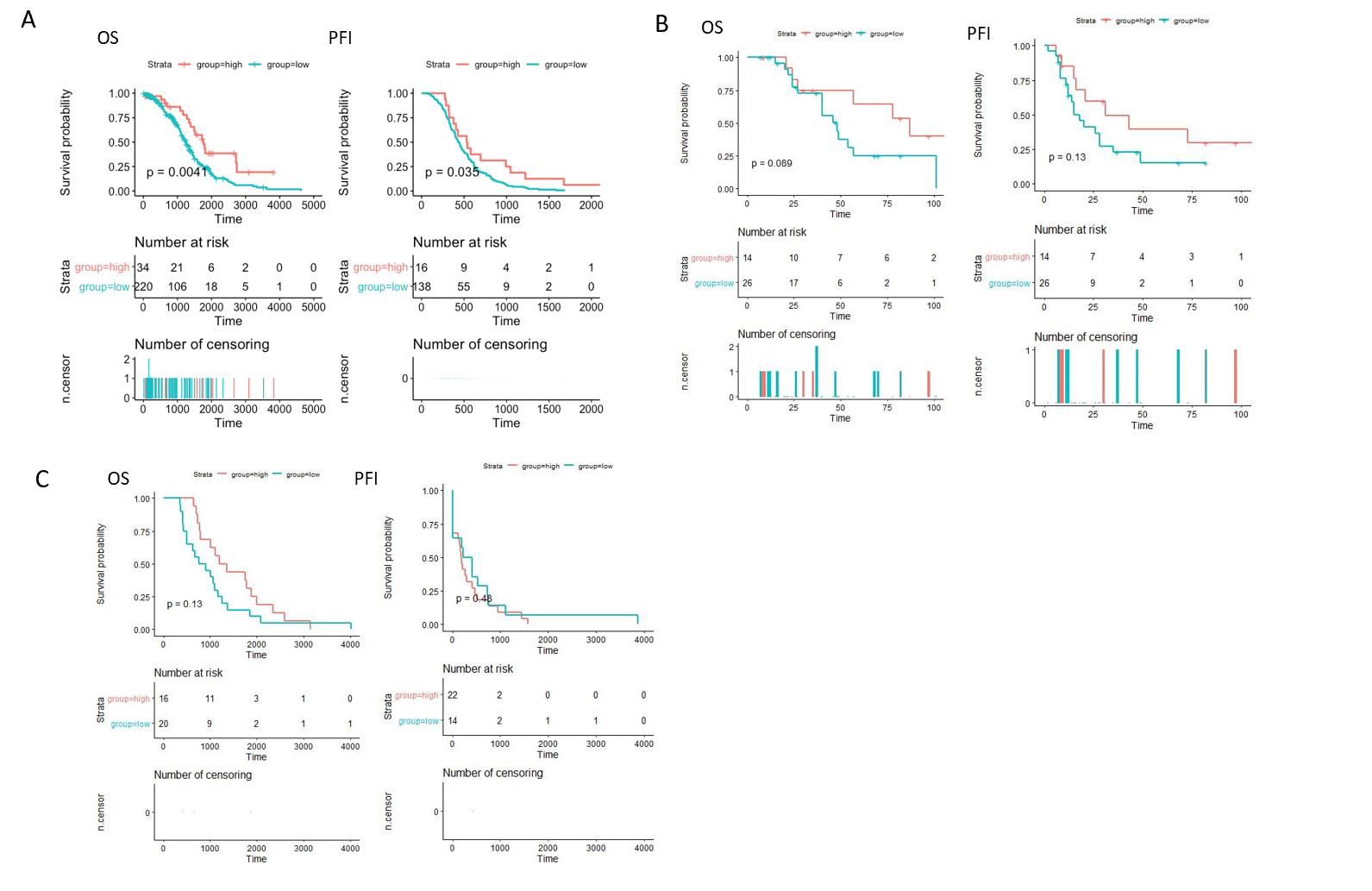
**

**Supplementary Figure 6.** Relationship of *ELP3* expression with survival outcome. (A) Overall survival (OS) and progression free interval (PFI) in the *ELP3* high and low expression groups in the TCGA-OV dataset. (B) Overall survival (OS) and progression free interval (PFI) in the *ELP3* high and low expression groups in the GSE32063 dataset. (C) Overall survival (OS) and progression free interval (PFI) in the *ELP3* high and low expression groups in the GSE63885 dataset. The numbers below the figures denote the number of patients at risk in each group.
